# Evaluation of Vitamin-D Status and Its Association with Clinical Outcomes Among COVID-19 Patients in Pakistan

**DOI:** 10.4269/ajtmh.21-0577

**Published:** 2021-11-10

**Authors:** Muhammad Sohaib Asghar, Farah Yasmin, Kartik Dapke, Syed Muhammad Ismail Shah, Muhammad Daim Bin Zafar, Anosh Aslam Khan, Osama Mohiuddin, Salim Surani

**Affiliations:** ^1^ Department of Internal Medicine, Dow University of Health Sciences–Ojha Campus, Karachi, Pakistan;; ^2^ Department of Internal Medicine, Dow University of Health Sciences, Karachi, Pakistan;; ^3^ Indira Gandhi Government Medical College, Nagpur, India;; ^4^ Department of Internal Medicine, Ziauddin Medical University, Karachi, Pakistan;; ^5^ Adjunct Clinical Professor of Medicine and Pharmacology, Texas A&M University, College Station, Texas

## Abstract

The risk of acute respiratory tract infections is particularly pronounced in patients deficient in 25-hydroxyvitamin D (25(OH)D). With respect to COVID-19, there are conflicting evidence on the association of 25(OH)D levels with disease severity. We undertook this study to evaluate the 25(OH)D status in COVID-19 patients admitted in Karachi, Pakistan, and associated vitamin D deficiency with primary outcomes of mortality, length of stay, intubation, and frequency of COVID-19 symptoms. A total of 91 patients were evaluated for 25(OH)D status during their COVID-19 disease course. 25-hydroxyvitamin D levels were classified as deficient (< 10 ng/mL), insufficient (10–30 ng/mL), or sufficient (> 30 ng/mL). The study population comprised 68.1% males (*N* = 62). The mean age was 52.6 ± 15.7 years. Vitamin D deficiency was significantly associated with intensive care unit (ICU) admission (RR: 3.20; *P* = 0.048), invasive ventilation (RR: 2.78; *P* = 0.043), persistent pulmonary infiltrates (RR: 7.58; *P* < 0.001), and death (RR: 2.98; *P* < 0.001) on univariate Cox regression. On multivariate Cox regression, only death (RR: 2.13; *P* = 0.046) and persistent pulmonary infiltrates (RR: 6.78; *P* = 0.009) remained significant after adjustment for confounding factors. On Kaplan Meier curves, vitamin D deficient patients had persistent pulmonary infiltrates and a greater probability of requiring mechanical ventilation than patients with 25(OH)D ≥ 10 ng/mL. Mechanical ventilation had to be initiated early in the deficient group during the 30-day hospital stay (Chi-square: 4.565, *P* = 0.033). Patients with 25(OH)D ≥ 10 ng/mL also demonstrated a higher probability of survival than those with 25(OH)D concentrations < 10 ng/mL. 25-hydroxyvitamin D deficient population had longer hospital stays and worse outcomes.

## INTRODUCTION

Vitamin D deficiency has been associated with a greater risk of respiratory tract infections.^1^ The receptors for 25(OH)D are expressed on macrophages (and dendritic cells) and are known to regulate the transcription process, including some genes encoding antimicrobial peptides and may play a role in warding off respiratory infections.^2^ 25-hydroxyvitamin D is also known to protect from free radical–mediated oxidative injury. In the renin-angiotensin pathway, 25(OH)D is known to promote the expression of the angiotensin-converting enzyme 2 (ACE-II), which has been shown to be downregulated by SARS-COV-2. Other mechanisms that vitamin D levels could impact the morbidity and mortality of SARS-COV-2 may include minimizing the pro-inflammatory response in these patients including selective separation of inflammatory cytokines; inducing the innate and acquired antiviral immune response and/or local conversion of 25(OH)D to 1,25-(OH)2D by an increase expression of CYP27B1 enzyme in lung epithelial cells. While there is conflicting evidence on the association of 25(OH)D levels with COVID-19 infection, it is a plausible association that warrants investigation. Szeto et al.^3^ found no significant association of 25(OH)D levels with primary outcomes like mortality, intubation, and renal replacement. Whereas Abrishami et al.^4^ found significant associations of 25(OH)D deficiency with decreased survival and increased total lung involvement. Hence, we undertook this study to evaluate the role of 25(OH)D status in COVID-19 patients, and the association of vitamin D deficiency with primary outcomes like mortality, length of stay, intubation, and frequency of COVID-19 symptoms in these patients.

## METHODS

A retrospective study was conducted between May and November 2020, for 6 months at a tertiary care hospital in Karachi, Pakistan. It is a private hospital and one of the largest referral units for COVID-19 in the city. Vitamin D status was evaluated in 91 patients during their COVID-19 disease course. The 25(OH)D concentrations were ascertained in these patients usually at admission by taking a serum sample volume of 10–12 µL for analysis by an automated kit method utilizing Electrochemiluminescence enzyme immunoassay (cobas e 411, Roche Diagnostics). Serum was collected using standard sampling tubes or tubes containing separating gel for this assay. 25-hydroxyvitamin D is stable for 8 hours at 20–25°C. Samples containing precipitates were centrifuged prior to measurement within 2 hours. Minimum detection limit is 3.0 ng/ml. COVID-19 infection was diagnosed from either nasopharyngeal or oropharyngeal swab using polymerase chain reaction (PCR). The diagnostic kit used the principle of real-time fluorescence (RT-PCR), USA-WA1/2020 stock concentration 2.8E+05 TCID50/mL, with a lower detection limit of 0.003 TCID50/mL. Serum 25(OH)D concentrations < 10 ng/mL levels were classified as deficient, 10–30 ng/mL as insufficient, and > 30 ng/mL as sufficient. To assess the role of 25(OH)D status in relation to the disease clinical features, all data were classified into two subgroups based on 25(OH)D that were less than or ≥ 10 ng/mL. The primary outcomes were mortality, length of hospital stay, intubation, and frequency of COVID-19 symptoms, and secondary outcomes were relationship with comorbidities, pulmonary infiltrates, and in-hospital events (like acute respiratory distress syndrome (ARDS), multi-organ failure [MODS], thrombotic event, intubation, etc.). Age, gender, body mass index (BMI), and outcome data was determined/extracted from patient review charts in the hospital’s electronic medical records. BMI was categorized as below or above 26 kg/m^2^ for high-risk population as guided by the World Health Organization.^5^ Simple linear regression was performed to determine the outcomes of various effect modifiers on 25(OH)D status, further univariate and multivariate Cox regression was conducted to obtain hazard ratio for significant variables. Kaplan Meier curves were constructed to evaluate the probability distribution of the 25(OH)D deficient group with mechanical ventilation, resolving of pulmonary infiltrates on chest X-ray, and survival during the hospital stay. A log-rank test was applied to determine the survival distributions between groups, and a *P* value of < 0.05 was considered statistically significant. All analysis was conducted using SPSS version 25.0 (IBM Corp, Armonk, NY) and variables were reported using descriptive statistics. Scatter plots were also generated accordingly for significant associations.

## RESULTS

Majority of our study population had insufficient 25(OH)D levels (Supplemental Figure 1). Overall 25(OH)D mean (SD) concentrations of all patients in this study were 21.4 (10.3) ng/mL with 20% (18/91) had deficient levels, 57% (52/91) had insufficient levels, while 23% (21/91) had sufficient levels. The study population comprised 68.1% males (*N* = 62). The mean (SD) age was 52.6 (15.7) years. Of the cases 69.2% were managed in isolation wards and the remaining in intensive care units (ICUs). In addition, 20.9% of patients died during hospital stay as demonstrated in Table 1. Based on cox univariate analysis, vitamin D deficient patients were more likely to be admitted to the ICU (HR: 3.20; *P* = 0.048), invasive ventilation (HR: 2.78; *P* = 0.043), persistent pulmonary infiltrates (HR: 7.58; *P* < 0.001), and death (HR: 2.98; *P* < 0.001) as shown in Table 2. On multivariate cox regression, only death (HR: 2.13; *P* = 0.046) and persistent pulmonary infiltrates (HR: 6.78; *P* = 0.009) remain significant after adjustment of confounding factors. The majority of the population presented with fever 79.12% (*N* = 72) and dry cough 75.82% (*N* = 69) as shown in Supplemental Table 1. There was a higher prevalence of symptoms among patients with 25(OH)D < 10 ng/mL in comparison to those with 25(OH)D ≥ 10 ng/mL, including cough with sputum (38.9% [*N* = 7/18] versus 13.7% [*N* = 10/73]; *P* = 0.022), dyspnea/shortness of breath (72.2% [13/18] versus 35.6% [26/73]; *P* = 0.005), fatigue (55.6% [10/18] versus 26.0% [19/73]; *P* = 0.016), nasal congestion/rhinorrhea (38.9% [7/18] versus 13.7% [10/73]; *P* = 0.022), diarrhea (38.9% [7/18] versus 15.1% [11/73]; *P* = 0.043), anosmia (22.2% [4/18] versus 5.5% [4/73]; *P* = 0.046), and malaise (50% [9/18] versus 17.8% [13/73]; *P* = 0.007). Patients with 25(OH)D < 10 ng/mL also exhibited increased length of hospital stay (10.61 ± 6.06 versus 7.36 ± 5.71 days; *P* = 0.040), were more likely to be intubated (33.3% [6/18] versus 6.8% [5/73]; *P* = 0.007), suffered from ARDS (22.2% [4/18] versus 4.1% [3/73]; *P* = 0.026) than those with 25(OH)D ≥ 10 ng/mL.

**Table 1:** Demographic and clinical characteristics of patients with respect to their mean 25-hydroxyvitamin D levels

Variables	*N* (%)	Mean 25-hydroxyvitamin D levels	*P* value
Age (years)			0.280
≤ 50	41 (45.1%)	22.93 ± 8.55	
> 50	50 (54.9%)	20.19 ± 11.53	
BMI (kg/m^2^)			0.235
< 26	48 (52.7%)	22.37 ± 8.85	
> 26	43 (47.2%)	20.38 ± 11.79	
Gender			0.136
Males	62 (68.1%)	22.51 ± 11.72	
Females	29 (31.9%)	19.11 ± 5.99	
Hospital stay			0.008
Isolation ward	63 (69.2%)	23.47 ± 8.67	
ICU	28 (30.8%)	16.82 ± 12.31	
Mode of ventilation			0.001
Invasive	11 (12.1%)	11.97 ± 8.22	
Noninvasive	80 (87.9%)	22.73 ± 9.95	
Clinical outcome			< 0.001
Recovered	72 (79.1%)	23.38 ± 9.07	
Death	19 (20.9%)	14.04 ± 11.67	
Resolving of pulmonary infiltrates			0.004
Yes	67 (73.6%)	23.39 ± 9.21	
No	24 (26.4%)	15.94 ± 11.48	
Seasonal variability			0.066
Admissions from May to August	47 (52.0%)	23.19 ± 8.75	
Admissions from September to November	44 (48.0%)	19.55 ± 11.60	

BMI = body mass index; ICU = intensive care unit. Mann Whitney U-test used to compute *P* values.

**Table 2:** Linear and Cox regression of 25-hydroxyvitamin D status with study variables

Linear regression of 25-hydroxyvitamin D status with study variables with 25-hydroxyvitamin D levels as dependent variable							
Variables	Unstandardized coefficients beta	95% Confidence interval				Standard error (SE)	*P* value
		Lower	Upper				
Ward vs. ICU stay	−6.65	−11.13	−2.17			2.25	0.004
Recoveries vs. death	−4.66	−7.14	−2.19			1.24	< 0.001
Resolving vs. persistence of pulmonary infiltrates	−7.45	−12.11	−2.80			2.34	0.002
Noninvasive vs. invasive ventilation	−10.76	−17.00	−4.51			3.14	0.001
Multivariable Cox regression with the outcome variable of 25-hydroxyvitamin D levels < 10 ng/mL							
	Univariate analysis				Multivariate analysis		
Variables	Hazard ratio	95% CI		*P* value	Hazard ratio	95% CI	*P* value
Isolation ward	1.00				1.00		
ICU stay	3.20	1.01–10.13		0.048	1.49	0.41–5.38	0.538
Recoveries	1.00				1.00		
Death	2.98	1.68–5.29		< 0.001	2.13	1.01–4.50	0.046
Persistent pulmonary infiltrates							
No	1.00				1.00		
Yes	7.57	2.60–22.04		< 0.001	6.78	1.61–28.54	0.009
Noninvasive ventilation	1.00				1.00		
Invasive ventilation	2.78	1.03–7.49		0.043	1.24	0.80–1.91	0.321

BMI = body mass index; DM = diabetes; HTN = hypertension; ICU = intensive care unit; IHD = ischemic heart disease. Multivariate analysis includes adjustment for age, gender, BMI, seasonality, comorbidities, and length of hospital stay.

**Figure 1.**
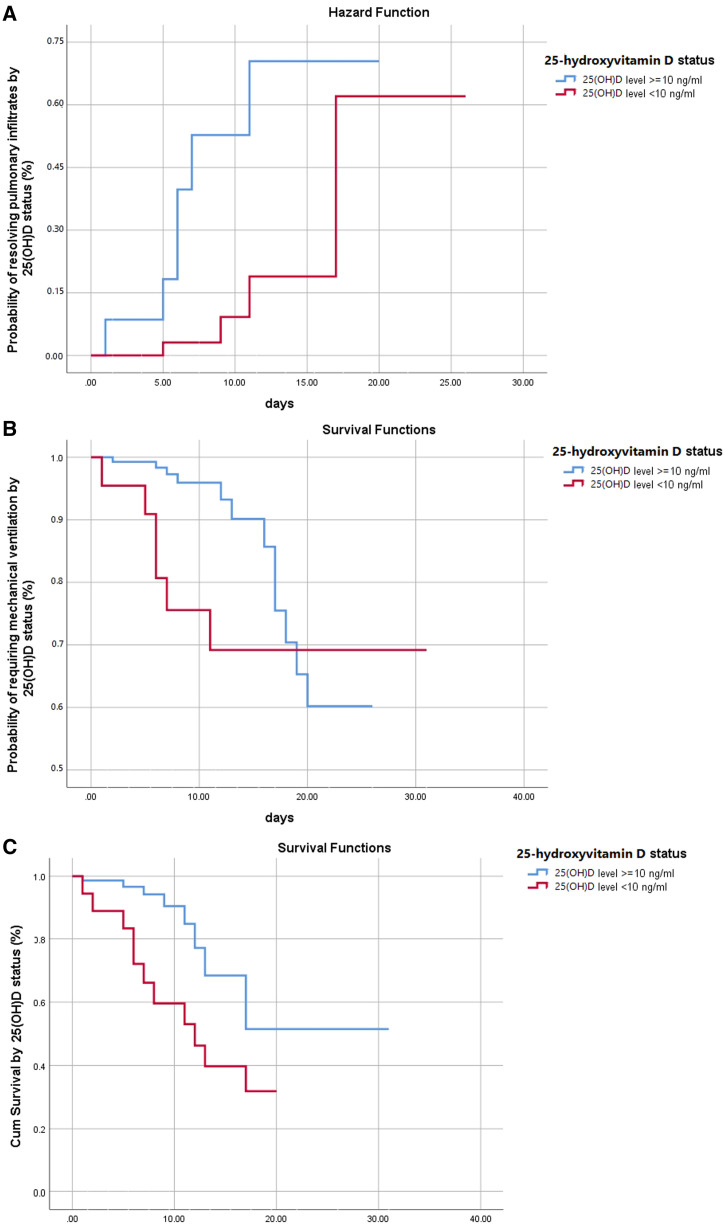
(**A**) Kaplan Meier curves of 25-hydroxyvitamin D deficiency with respect to resolving of pulmonary infiltrates, (**B**) mechanical ventilation, and (**C**) survival during 30-day hospital stay. This figure appears in color at www.ajtmh.org.

**Figure 2.**
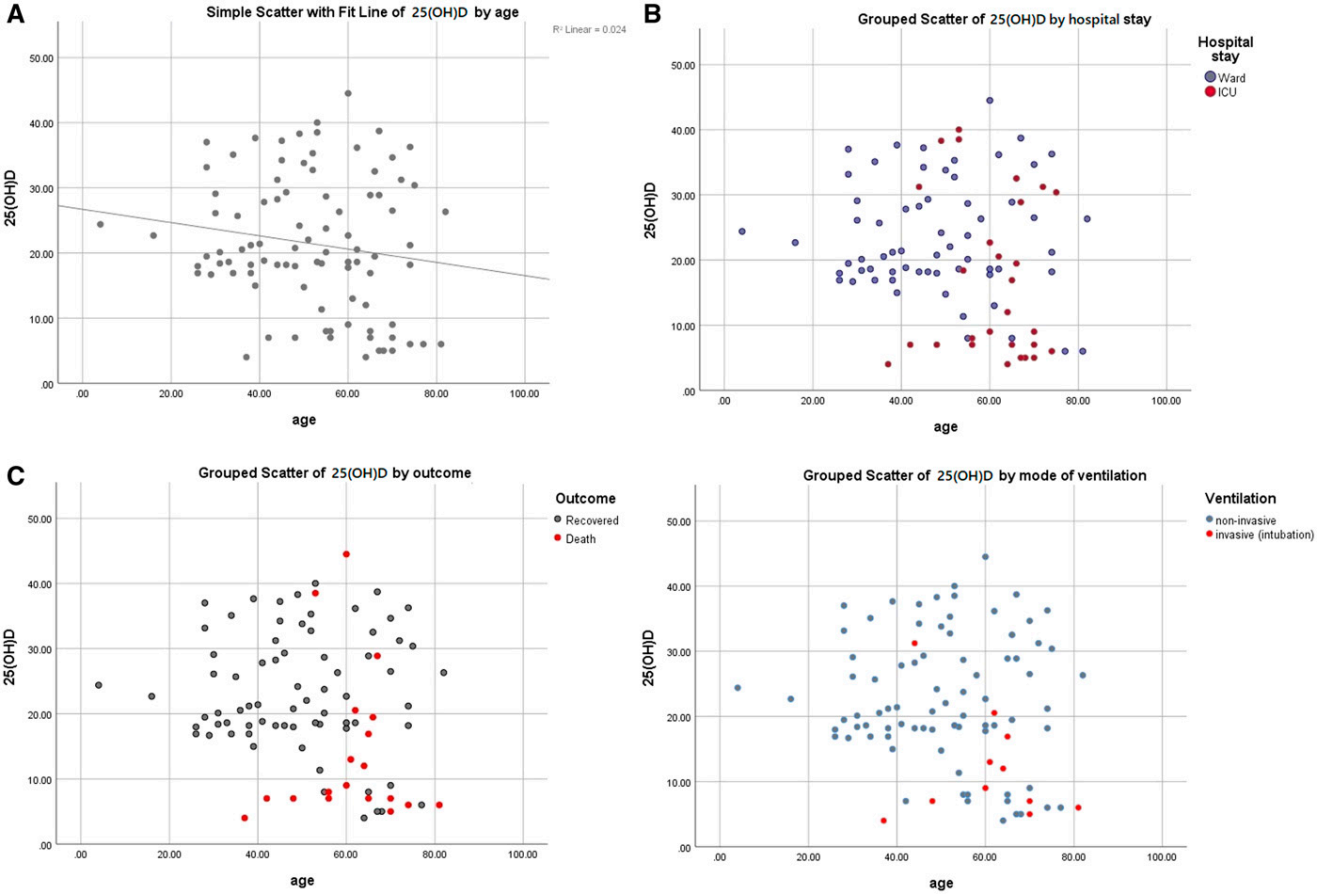
(**A**) Scatter plots of 25-hydroxyvitamin D levels with respect to age (**B**) along with hospital stay, (**C**) clinical outcome, and (**D**) mode of ventilation. This figure appears in color at www.ajtmh.org.

Figure 1 depicts the Kaplan Meier curves of 25(OH)D deficiency concerning invasive ventilation, resolving of pulmonary infiltrates, and survival during a 30-day hospital stay. A significant difference was observed when compared with 25(OH)D ≥ 10 ng/mL and deficient group (Chi-square: 4.763, *P* = 0.029) for resolving pulmonary infiltrates (Figure 1A). 25(OH)D deficient patients (< 10 ng/mL) had a greater probability of requiring mechanical ventilation than patients with 25(OH)D ≥ 10 ng/mL. Mechanical ventilation had to be initiated early in the deficient group during the 30-day hospital stay (Chi-square: 4.565, *P* = 0.033) (Figure 1B). Patients with 25(OH)D levels ≥ 10 ng/mL also demonstrated higher probability of survival than those with 25(OH)D levels < 10 ng/mL (Chi-square: 6.365, *P* = 0.012) (Figure 1C). Figure 2 demonstrates the association of 25(OH)D status with age and their correlations with inpatient mortality, ICU stays, and invasive ventilation required because of COVID-19 infection. A scatter plot relating serum 25(OH)D levels with age showed a declining trend. A weakly negative correlation is established, that is, as the age increases, 25(OH)D status may decrease slightly (R^2 ^= 0.024) as shown in Figure 2A. The number of red dots in Figure 2B represents ICU stay, which is significantly more in patients having 25(OH)D levels < 10 ng/mL. Also, the trend inpatient mortality is observed more for lower levels of 25(OH)D as shown in Figure 2C. While a majority recovered on noninvasive ventilation, a small proportion of patients with deficient 25(OH)D warranted invasive ventilation as shown in Figure 2D.

## DISCUSSION

It has been documented that 25(OH)D plays a vital role in the regulation of the renin-angiotensin system. SARS-CoV-2 is known to exploit the ACE-II receptors to facilitate its entry into the host cell.^6^ 25(OH)D modulates multiple immune mechanisms to contain the virus including dampening of viral entry and replication of SARS-CoV-2, suppressing the hyperinflammatory state by increasing anti-inflammatory cytokine levels.^7^^,^^8^ One major limitation of the study is the small sample size since the study was conducted at a single institution. The possibility of collider bias due to convenience sampling cannot be ruled out. Hence, our findings limit generalizability and power. Another major limitation of this study is the additional confounders that may account for the differences in 25(OH)D status and outcomes, which impacts the validity of the findings. For instance, reduced outdoor activities may be impacted due to illness and also cause lower vitamin D status. Similarly, seasonal variation, age, pregnancy, and thyroid function may influence circulating vitamin D levels. Small sample size will further reduce the power to detect a significant difference. Nevertheless, our results suggest that 25(OH)D deficiency might be linked to aggravation of symptoms, invasive interventions, ICU stays, hence reducing mortality. Sufficient vitamin D levels were also associated with survival promotion and quicker recovery. There is a potential for reverse causality to explain these findings, as inflammation may dysregulate vitamin D metabolism because 25(OH)D is a negative acute-phase reactant.^9^ Another similar association is that of a prothrombotic state, seen in vitamin D deficiency as well as COVID-19 infection. If 25(OH)D is functional in reducing the severity of COVID-19, it is wise to supplement the treatment regimen with vitamin D to decrease the impact of the pandemic.^10^ However, studies have not supported supplementation of vitamin D because of no difference in mortality, need for intubation, and length of hospital stay.^11^^,^^12^ Not only COVID-19 positivity is associated with deficient 25(OH) D levels,^13^ worse morbidity outcomes in older age group are reportedly more likely to be vitamin D deficient.^14^ A study conducted 330 patients found no significant associations of 25(OH)D levels with body mass index (BMI) similar to our findings, but also with duration of stay, oxygen requirements, and death opposing our findings.^15^ Another study on 73 patients concluded lower levels of 25(OH)D observed in deceased patients as compared with discharged patients, while lesser involvement of lungs on computed tomography (CT) scan was observed in those with sufficient 25(OH)D levels.^4^ Lastly, the study conducted by Szeto et al.^3^ found those with 25(OH)D deficiency did not differ from those who had sufficient levels in terms of gender, BMI, or comorbidities, which correlated with our findings. However, clinical outcomes like mortality, intubation, and length of stay were not found associated with opposing our results.^3^

## CONCLUSION

25-hydroxyvitamin D–deficient population had longer hospital stays, slower recovery, and worse outcomes. The clinical presentation also differed significantly in the deficient population. Patients with sufficient 25(OH)D levels had a lesser incidence of invasive interventions, complications, and death, pointing toward a possible beneficial effect of vitamin D on the immune system. It is wise on part of the health care providers to adopt a cautious approach when treating COVID-19 patients with depleted 25(OH)D levels till randomized clinical trials provide sufficient evidence supporting the association.

## Supplemental tables


Supplemental materials
