# Supplementary material for: Evaluation of Vitamin-D Status and Its Association with Clinical Outcomes Among COVID-19 Patients in Pakistan
Source: Am J Trop Med Hyg. 2021 Nov 10;106(1):150–5. doi: 10.4269/ajtmh.21-0577 (PMC8733525; doi:10.4269/ajtmh.21-0577)
Supplement: Supplementary file 1 [file tpmd210577.SD1.pdf]

Supplemental Table 1: Clinical characteristics of the study population with respect to 25-hydroxyvitamin D status (n=91).					
S.no	**Variables**	**Characteristics**	**25(OH)D >= 10 ng/ml (n=73)**	**25(OH)D < 10 ng/ml (n=18)**	**p-value**
1	Comorbidities	Diabetes	28 (38.4%)	6 (33.3%)	0.693*
		Hypertension	27 (37.0%)	9 (50.0%)	0.312*
		IHD	3 (4.1%)	2 (11.1%)	0.256**
		CVA	2 (2.7%)	1 (5.6%)	0.488**
		CKD	1 (1.4%)	2 (11.1%)	0.099**
		CLD	2 (2.7%)	2 (11.1%)	0.174**
		COPD	1 (1.4%)	1 (5.6%)	0.358**
		Asthma	2 (2.7%)	0 (0.0%)	0.037**
		Hypothyroidism	0 (0.0%)	1 (5.6%)	0.198**
		Atrial fibrillation	1 (1.4%)	0 (0.0%)	1.000**
2	Symptomatology	Fever	60 (82.2%)	12 (66.7%)	0.194**
		Chills/Rigors	4 (5.5%)	3 (16.7%)	0.136**
		Cough – Dry	56 (76.7%)	13 (72.2%)	0.761**
		Cough – with Sputum	10 (13.7%)	7 (38.9%)	0.022**
		Sore Throat/Throat Pain	16 (21.9%)	6 (33.3%)	0.360**
		Chest Pain/Tightness	4 (5.5%)	2 (11.1%)	0.594**
		Dyspnea/Shortness of Breath	26 (35.6%)	13 (72.2%)	0.005*
		Fatigue	19 (26.0%)	10 (55.6%)	0.016*
		Nasal Congestion/Rhinorrhea	10 (13.7%)	7 (38.9%)	0.022**
		Headache	18 (24.7%)	2 (11.1%)	0.342**
		Anorexia – Loss of Appetite	6 (8.2%)	3 (16.7%)	0.373**
		Arthralgia/Myalgia	12 (16.4%)	3 (16.7%)	1.000**
		Vomiting	8 (11.0%)	3 (16.7%)	0.450**
		Nausea	14 (19.2%)	4 (22.2%)	0.749**
		Diarrhea	11 (15.1%)	7 (38.9%)	0.043**
		Abdominal Pain	6 (8.2%)	4 (22.2%)	0.105**
		Ageusia- Loss of Taste sense	2 (2.7%)	1 (5.6%)	0.488**
		Anosmia- Loss of Smell sense	4 (5.5%)	4 (22.2%)	0.046**
		Confusion/Delirium	3 (4.1%)	3 (16.7%)	0.089**
		Conjunctival Congestion	3 (4.1%)	0 (0.0%)	0.611**

		Dizziness/Lightheadedness	5 (6.8%)	3 (16.7%)	0.348**
		Hemoptysis	1 (1.4%)	0 (0.0%)	1.000**
		Malaise	13 (17.8%)	9 (50.0%)	0.007**
		Night Sweat	1 (1.4%)	1 (5.6%)	0.358**
		Sneezing	4 (5.5%)	2 (11.1%)	0.594**
		Rash	1 (1.4%)	2 (11.1%)	0.099**
		Swollen Neck Glands/Lymphadenopathy	1 (1.4%)	1 (5.6%)	0.358**
		Seizure	0 (0.0%)	0 (0.0%)	-
3	In-hospital events	Length of hospital stay (mean ± SD)	7.36 ± 5.71	10.61 ± 6.06	0.040***
		Intubated	5 (6.8%)	6 (33.3%)	0.007**
		Mean duration of intubation (in days)	7.14 ± 3.25	8.07 ± 4.96	0.458***
		Bilateral pulmonary infiltrates	24 (32.9%)	18 (44.4%)	0.357*
		ARDS	3 (4.1%)	4 (22.2%)	0.026**
		Multi-organ failure (MODS)	5 (6.8%)	4 (22.2%)	0.072**
		Thrombotic events	7 (9.6%)	4 (22.2%)	0.218**

* chi-square test
** Fisher's exact test
*** Mann-Whitney U test
IHD: ischemic heart disease; CVA: cerebrovascular accident; COPD: chronic obstructive pulmonary disease; ARDS: acute respiratory distress syndrome; CLD: chronic liver disease; CKD: chronic kidney disease; 25(OH)D: 25-hydroxyvitamin D.

Supplemental figure legend:
Supplementary Figure 1: The 25-hydroxyvitamin D status in study population (Histogram).

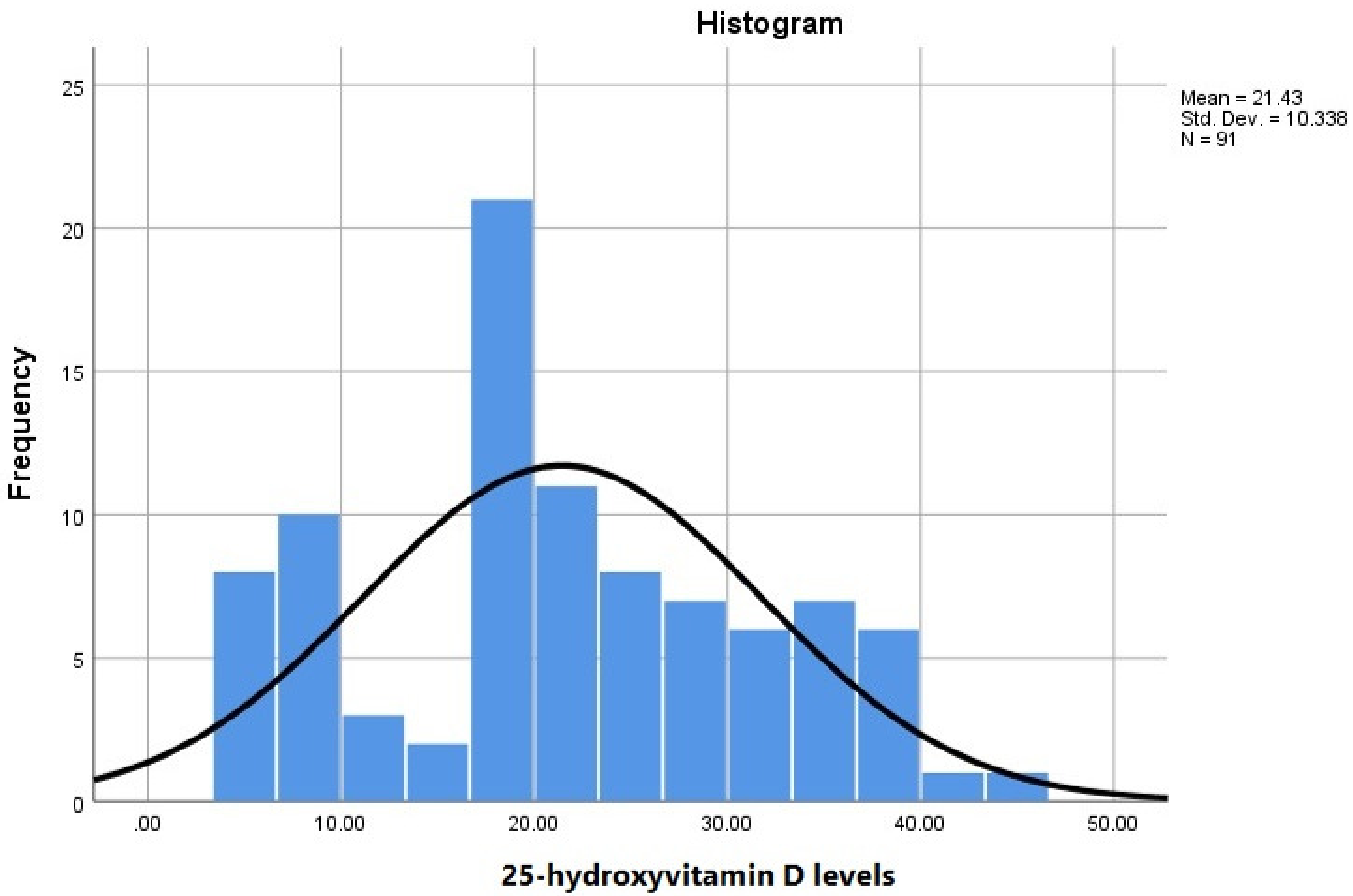

Histogram
Mean = 21.43
Std. Dev. = 10.338
N = 91
Frequency
25
20
15
10
5
0
.00
10.00
20.00
30.00
40.00
50.00
25-hydroxyvitamin D levels
